# Quantification of the Diversity in Gene Structures Using the Principles of Polarization Mapping

**DOI:** 10.3390/cimb45020111

**Published:** 2023-02-18

**Authors:** Dmitry Zimnyakov, Marina Alonova, Anatoly Skripal, Sergey Dobdin, Valentina Feodorova

**Affiliations:** 1 Physics Department, Yury Gagarin State Technical University of Saratov, 77 Polytechnicheskaya St., 410054 Saratov, Russia; 2 Precision Mechanics and Control Institute of Russian Academy of Sciences, 24 Rabochaya St., 410024 Saratov, Russia; 3 Institute of Physics, Saratov State University, 83 Astrakhanskaya St., 410012 Saratov, Russia

**Keywords:** bioinformatics, nucleotide sequences, polarization encoding, Stokes vector components, visualization, coronavirus genome

## Abstract

Results of computational analysis and visualization of differences in gene structures using polarization coding are presented. A two-dimensional phase screen, where each element of which corresponds to a specific basic nucleotide (adenine, cytosine, guanine, or thymine), displays the analyzed nucleotide sequence. Readout of the screen with a coherent beam characterized by a given polarization state forms a diffracted light field with a local polarization structure that is unique for the analyzed nucleotide sequence. This unique structure is described by spatial distributions of local values of the Stokes vector components. Analysis of these distributions allows the comparison of nucleotide sequences for different strains of pathogenic microorganisms and frequency analysis of the sequences. The possibilities of this polarization-based technique are illustrated by the model data obtained from a comparative analysis of the spike protein gene sequences for three different model variants (Wuhan, Delta, and Omicron) of the SARS-CoV-2 virus. Various modifications of polarization encoding and analysis of gene structures and a possibility for instrumental implementation of the proposed method are discussed.

## 1. Introduction

Further development of techniques for rapid testing and identification of pathogen microorganisms, causative agents of either human or animal infectious diseases, relates to the design and verification of novel efficient approaches based on the principles of bioinformatics. In recent years, the emergence of new infections and the increasing frequency of outbreaks of viral infectious diseases with a high epidemic or even pandemic potential are associated with identification of viral mutants. This requires a detailed study of the biodiversity of the corresponding pathogenic microorganisms. Accordingly, this stimulates a search for efficient technologies relating to rapid and accurate polymorphism analysis of the target genes. Over the past half century, the basic principles and approaches to the analysis of genetic diversity of living organisms have been developed. Current advances in this area are the result of abundance of research works in the fields of molecular biology and bioinformatics.

Analysis of the genetic structure of biological objects includes two basic stages. In the first stage, the relevant DNA is sequenced using one of two well-established techniques known as next-generation sequencing (NGS) technology: short-read and long-read sequencing [1]. After the first stage, the primary structure of a linear biomolecule of a certain length is determined by indicating the positions of four basic nucleotides (adenine (A), cytosine (C), thymine (T), and guanine (G)) in the target DNA. In the second essential stage, the structure of the obtained (A,C,T,G) sequences is analyzed to identify the features characterizing the studied DNA.

Note that this secondary-stage analysis is no less important than the initial DNA sequencing and plays a decisive role in many bioinformatics applications. It is usually performed using a variety of software techniques for processing the nucleotide-corresponding symbols and their groups in the (A,C,T,G) sequences obtained in the first stage.

Due to the quasi-random nature of the distributions of the four basic symbols in the string-like structures obtained by sequencing, these computerized methods for analysis, visualization, and simulation of the nucleotide sequences are based on the fundamental principles of mathematical statistics, information theory, and theory of random processes. In particular, algorithms for the analysis and modeling of DNA-associated symbol sequences, based on the method of Bayes prediction [2], features of Markov [3,4] or Jukes-Cantor [5] processes, the Monte–Carlo method [6], specially designed probabilistic models [7], and multidimensional scaling and clustering techniques [8,9] have been successfully applied in bioinformatics.

An inherent property of all gene-associated symbol sequences is their complexity, which can be quantified in several ways. One of the most popular approaches to measuring the complexity of a symbol sequence of finite length is the application of the Ziv-Lempel complexity measure [10]. The minimal number of steps required for the sequence synthesis defines this measure. In this synthesis, two operations are allowed at each step, such as the generation of a new symbol or the copying of a fragment from the already synthesized part of the sequence. When applied to the analysis of gene-based symbol sequences, the Ziv–Lempel measure is not free from shortcomings. In particular, it does not take into account the occurrence of isomorphic repeats in the analyzed sequence. The generalized Ziv–Lempel approach considered in [11] is free from this disadvantage.

In addition to complexity measures based on the Ziv–Lempel algorithm, the measure of linguistic complexity is often used in bioinformatics [12,13]. The linguistic complexity of one-dimensional sequences is introduced in terms of vocabulary usage. This is the ratio of the actual vocabulary of words (symbol combinations) of a given length $L_{W}$ to the maximal possible vocabulary for the given sequence. The linguistic complexity is a product of all vocabulary usages for $1\leq L_{W}\leq N_{t}-1$ ($N_{t}$ is the number of symbols in the sequence).

Computational analysis of long-range correlations in the positions of symbol groups in the sequences can be carried out by calculating the so-called Hurst exponent [14]. Depending on its value, this parameter characterizes the presence (“persistence”) or absence (“anti-persistence”) of long-range correlations in the symbol positions. A comparative analysis of the Ziv–Lempel complexity measures and the Hurst exponents for model DNA sequences showed that the complexity of introns and regulatory regions is lower than that of coding regions, whilst the Hurst exponent is larger.

Beginning with a pioneering work by C.-K. Peng et al. [15], the existence of large-scale correlations between the positions of nucleotide groups in DNA sequences gives grounds to consider their properties in terms of “fractality”. The Hurst exponent is one of the key parameters applied within the framework of the fractal approach. In addition, a number of so-called critical exponents can be introduced for the quantification of various features in the fractal structure of DNA sequences [16].

Computation methods for visualization of the structure of DNA-associated symbol sequences, developed and implemented since the early nineties of the last century, are based on several popular algorithmic approaches. Among these approaches, the sequence logos [17] and chaos game representation (CGR) [18] should be mentioned. The CGR technique in its original form, proposed by H. Jeffrey, allows for the synthesis of two-dimensional sequence-associated patterns. It is based on the application of the system of two linear iterative functions to generate sequential (x, y) coordinates of the points in the synthesized pattern. These points are associated with consecutive (A,C,T,G) symbols in the patterned sequence. Since the pioneering work of H. Jeffrey, the CGR method has undergone further development; the current state-of-art and recent applications of this technique in bioinformatics have been reviewed in [19]. Among the numerous applications of CGR patterns for the analysis of DNA sequences, it is necessary to note the works devoted to the phylogenetic analysis of coronavirus sequences [20] and the classification of intra-coronavirus sequences [21]. In the latter case, the CGR technique was combined with an artificial neural network.

An original approach to the analysis of synthesized CGR patterns of DNA sequences was considered in [22]. It is based on a computer simulation of small-angle light scattering by a synthesized pattern. It was shown that the simulated small-angle-scattering data give a possibility to quantify the fractal properties of the analyzed CGR pattern. In turn, these properties can be used for the identification of complex hierarchical DNA sequences.

In addition to 2D imaging (mapping) of DNA-associated symbol sequences, the algorithms for representing genetic data in high-dimensional spaces (with D from 3 to 6) have also been developed over the past three decades [23]. Among the variety of such algorithms, 3D visualization techniques are of particular interest due to their sufficient visual evidence. A rendered symbol sequence is associated with a polygonal line as a set of points in a 3-dimensional Cartesian space. In particular, the H- [24], Z- [25], RY-, MK-, SW- [26], and C-curve [27] approaches to 3D visualization of genetic data were proposed. These approaches differ in the rules of transforming (A,C,T,G) symbol values and their positions in sequences into (x, y, z) coordinates of the points belonging to representing polygonal lines.

Thus, it can be assumed that such an abundance of computational methods for analysis, visualization, and modeling of DNA-associated character sequences largely provides a solution to the majority of bioinformatics problems. At the same time, the problem of gene differences analysis and visualization of these differences can also be solved using instrumental and instrumental–computational (hybrid) approaches. These approaches can be based on the principles of coherent optical and polarization analysis of quasi-random structures. At first glance, the development and implementation of such instrumental and hybrid methods with the existing abundance of efficient computational technologies for the analysis and visual representation of gene sequences seem redundant and far-fetched. Nevertheless, such activities may be of interest for further development of bioinformatics methods in terms of the introduction and application of new integral criteria for the similarity and diversity of gene sequences and the visualization of these features. In addition, mathematical representation of physical principles of coherent optical and polarization analysis and visualization of quasi-random structures can be used as the basis for new effective algorithms of computer solutions to bioinformatics problems.

Previously, a coherent optical technique was proposed to display unique genetic information in the form of the so-called gene-based speckle patterns (GB speckles, [28]). Within the framework of this technique, a sufficiently long fragment of an analyzed sequence of nucleotide triplets is converted into a square matrix with the size of N×N. Accordingly, the number of triplets in the chosen fragment should be equal to $N^{2}$. A composed matrix with the elements corresponding to various threefold combinations of four letters (A,C,T,G) is transformed to the numerical form using the following conversion rule: $X_{i,j}=16\Xi_{1}+4\Xi_{2}+\Xi_{3}-21$. Here, each of the three factors $\Xi_{1},\Xi_{2},\Xi_{3}$ takes the value from 1 to 4 in accordance with the following associations: A↔1,C↔2,G↔3,T↔4, and the lower indices “1–3” correspond to the position of a nucleotide in the triplet. The indices (i,j) (1≤i≤N,1≤j≤N) define the position of the given element in the matrix. Accordingly, the maximal value of 63 corresponds to TTT triplets, and the zero matrix elements are associated with AAA triplets. The synthesized matrix is considered as the basis for creating a random phase screen (i.e., a spatial light modulator with a random structure). Each element is multiplied by a certain phase factor $K_{\varphi }$: $X_{i,j}\to K_{\varphi }\cdot X_{i,j}$. The matrix is considered as a two-dimensional transparent structure of N×N elements. Each element, when the light beam passes through the structure, introduces a certain local phase delay $K_{\varphi }\cdot X_{i,j}$ to the propagating light wave. Being illuminated by a plane coherent light wave, such a gene-based phase screen produces a random spatial distribution of the transmitted light field in the far diffraction zone. Similar random distributions are known as speckle patterns [29]. In the discussed case, the randomness of the speckle pattern is caused by a close-to-random distribution of $K_{\varphi }\cdot X_{i,j}$ values across the synthesized matrix. At the same time, being a stochastic object, the microscopic structure of the formed speckle pattern is unique, representing a “fingerprint” of the analyzed gene sequence. A unique correspondence between an object and a diffracted coherent light field makes it possible, for example, to provide a holographic recording and coherent optical recognition of images.

Mutational changes in the analyzed nucleotide sequence inevitably leads to a spatial decorrelation of the formed gene-based speckle pattern with respect to the original pattern corresponding to an unchanged reference sequence. In accordance with [28], this feature can be used to identify important differences in nucleotide sequences corresponding to different strains of the specific biological object. The results of modeling this approach to characterize the diversity in nucleotide sequences showed an acceptable performance of the GB speckle technique in solving such problems. However, simple estimates of sensitivity relating to the correlation analysis of the GB speckle intensity to minor changes in the structure of the analyzed sequence (when replacing either one or two nucleotides) do not give encouraging results. In fact, a change in a single element of the phase screen matrix consisting of several hundred elements will lead to subtle changes in the amplitude and phase of the diffracted light field. In addition, the approach proposed in [28] to identify the changes in gene structures is based on the principle of coherent optical recognition with the use of a synthesized matched filter. This principle is difficult for instrumental implementation and overly sensitive to external influences.

At the same time, another approach to coherent optical characterization of diversity in nucleotide sequences is possible. This approach can also be based on the principle of representing genetic information in the form of quasi-random phase-modulating 2D structures. However, the readout principle may assume not a scalar but rather a vector approach to the analysis of the light field diffracted by a gene-associated phase-modulating structure. Accordingly, such a coherent optical approach should be based on polarization modulation of the readout coherent beam by this structure. The second essential stage is analysis of the local polarization structure in the diffracted light field. The goal of this work is to develop the basic principles of polarization visualization and quantification of differences in the gene structures and consider the advantages and pitfalls of this technique as a possible tool for application in bioinformatics.

## 2. Materials and Methods

### 2.1. Synthesis of a Gene-Based Virtual Phase Retarder

Let us consider the synthesis of a virtual multi-element polarization modulator corresponding to the analyzed nucleotide sequence. Beginning from a start codon, a sequence fragment is selected that includes $N=n^{2}$ nucleotide triplets (here, n is an integer value that satisfies the criterion $n^{2}\leq \left\lfloor N_{t}/3\right\rfloor$, where $N_{t}$ is the total number of nucleotides in the sequence). Each triplet in the selected fragment is assigned the submatrix ${\left(a_{ij}\right)}_{2\times 2}$ in accordance with the following rules:

(1) The positions of the submatrix elements correspond to certain items from the set of four basic nucleotides (A,C,G,T) (e.g., i=0,j=0→A, i=0,j=1→C, i=1,j=0→G, i=1,j=1→T);

(2) The value of an element determines the content of the corresponding nucleotide in the triplet and, accordingly, is in the range from 0 to 3. In addition, the sum of submatrix elements is always equal to 3.

As an example, consider this coding procedure for a portion of the nucleotide sequence for the gene B602L of the African swine fever (ASF) virus HuB20 strain (Acc. No. in the GenBank MW521382.1) [30]:

(1) $$\begin{array}{l} ATGGCATCAGGAGGA.........\\ \;\;\;\;\;⇓\\ \left(\begin{array}{cc} 1 & 0\\ 1 & 1 \end{array}\right)\left(\begin{array}{cc} 1 & 1\\ 1 & 0 \end{array}\right)\left(\begin{array}{cc} 1 & 1\\ 0 & 1 \end{array}\right)\left(\begin{array}{cc} 1 & 0\\ 2 & 0 \end{array}\right)\left(\begin{array}{cc} 1 & 0\\ 2 & 0 \end{array}\right)........ \end{array}$$

Note that the choice of assignment for the positions of submatrix elements to the basic nucleotides is arbitrary; only the sameness of this assignment is required during encoding of the sequence.

In the next step, a virtual phase screen is generated by sequentially combining N submatrices into the matrix structure ${\left(a_{ij}\right)}_{2n\times 2n}$ and multiplying each element by the scaling phase factor $K_{\varphi }$:

(2) $${\left(\Delta \varphi_{ij}\right)}_{2n\times 2n}=K_{\varphi }{\left(a_{ij}\right)}_{2n\times 2n}$$

Considering the synthesized phase screen as a multi-element phase retarder, we can assume that the encoded information is read by a plane coherent light wave, which is a superposition of x- and y-polarized plane waves with a certain phase delay between them. This approach opens a variety of possibilities in terms of polarization modulation of the readout light field by the synthesized gene-based structure. In the general case, the law of phase modulation of the x- and y-components of the readout field can be presented as:

(3) $$\begin{array}{l} {\left(\Delta \varphi_{ij}\right)}_{2n\times 2n}^{x}=K_{\varphi }^{x}\left(i,j\right)\cdot {\left(a_{ij}\right)}_{2n\times 2n};\\ {\left(\Delta \varphi_{ij}\right)}_{2n\times 2n}^{y}=K_{\varphi }^{y}\left(i,j\right)\cdot {\left(a_{ij}\right)}_{2n\times 2n}+f\left(i,j\right), \end{array}$$

where the function f(i,j) determines the modulation law of phase retardation for each element of the synthesized phase screen. The simplest case corresponds to a uniform phase retardation and the same phase shift of the x- and y-components of the readout light field:

(4) $$\left\{ \begin{array}{l} f\left(i,j\right)=const=K_{r};\\ K_{\varphi }^{x}\left(i,j\right)=K_{\varphi }^{y}\left(i,j\right)=const=K_{\varphi }. \end{array}\right.$$

### 2.2. Polarization Imaging of the Analyzed Nucleotide Sequences

**Figure 1:**
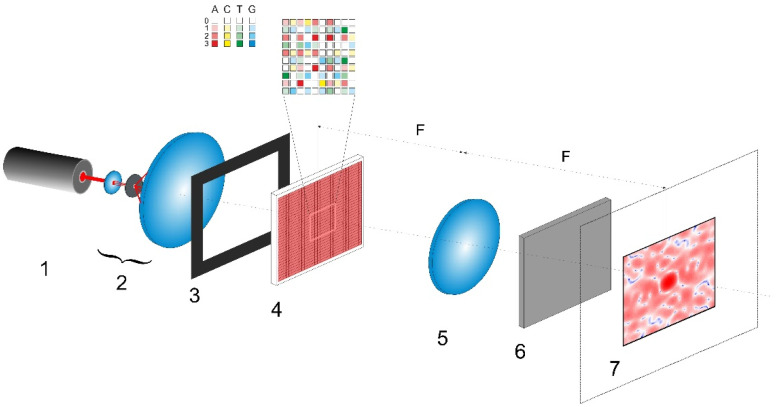
The scheme of speckle polarization mapping of a nucleotide sequence. 1—continuous-wave laser source; 2—telescopic system with a pinhole as a beam cleaner and expander; 3—aperture diaphragm; 4—gene-based spatial light modulator; 5—Fourier-transforming lens; 6—polarimetric unit; 7—output plane. A four-color presentation is used to display the principle of the content-to-phase transformation; the color saturation corresponds to the content of the given nucleotide in a coded triplet. White light indicates the absence of the given nucleotide.

The considered scheme for polarization imaging of encoded nucleotide sequences is presented in Figure 1. The synthesized phase screen ${\left(\Delta \varphi_{ij}\right)}_{2n\times 2n}$ (item 1) is located in the front focal plane of the Fourier-transforming lens (item 5) and is illuminated by a linearly polarized collimated light beam. The polarization plane of the readout beam is inclined at the angle of π/4 to the sides of the phase screen. The diffraction pattern is observed in the rear focal plane of lens 5; due to the quasi-random nature of the synthesized phase screen, this pattern will be speckle-modulated. At each point in the pattern, there will be a certain local polarization state of the diffracted readout field. These states are formed due to superposition of the incoming diffracted x- and y-polarized waves with different phase shifts. The local polarization states can be examined using a polarization analyzer (item 6) consisting of a rotating polarizer and a retarder plate and located directly behind the Fourier-transforming lens. Each local polarization state can be quantitatively described by a set of four components of the Stokes vector (see, e.g., [31]). These components are calculated from the amplitudes of the x- and y-components of the diffraction field and the phase shift between them at a given point.

Orthogonally polarized components of the diffraction field can be calculated using the Fourier transforms of the corresponding components in the boundary light field. This boundary field is formed directly behind the synthesized phase screen. Accordingly, this is described by the following set of formulas (see, e.g., [32]):

(5) $$E_{k,m}^{x,y}=\frac{1}{4N^{2}}\sum_{i=-N}^{N-1}\sum_{j=-N}^{N-1}\exp\left[-\tilde{j}\cdot K_{sc}\cdot \left\{\left(\pi /N\right)\left(k\cdot i+m\cdot j\right)-\Delta \varphi_{i,j}^{x,y}\right\}\right];$$

(6) $$\left\{ \begin{array}{l} s_{k,m}^{0}=\left({\left|E_{k,m}^{x}\right|}^{2}+{\left|E_{k,m}^{y}\right|}^{2}\right)/2;\\ s_{k,m}^{1}=\left({\left|E_{k,m}^{x}\right|}^{2}-{\left|E_{k,m}^{y}\right|}^{2}\right)/2s_{k,m}^{0};\\ s_{k,m}^{2}=2\left|E_{k,m}^{x}\right|\left|E_{k,m}^{y}\right|\cos\left(\delta_{k,m}\right)/2s_{k,m}^{0};\\ s_{k,m}^{3}=2\left|E_{k,m}^{x}\right|\left|E_{k,m}^{y}\right|\sin\left(\delta_{k,m}\right)/2s_{k,m}^{0}. \end{array}\right.$$

Here $\tilde{j}$ is the imaginary unit and we assume that amplitudes of the x- and y-components of the readout beam falling onto the phase screen are equal to 1. The indices k,m correspond to discrete coordinates in the diffraction plane. The scale factor $0<K_{sc}\leq 0.5$ is used to choose an area of interest in the diffraction plane (item 7 in Figure 1). The extreme value of 0.5 is determined by the condition of the absence of the aliasing effect in the analyzed spatial spectrum of the diffracted readout beam.

Note that $K_{sc}$ at the given value of N characterizes magnification of the formed diffraction pattern in plane 7 (Figure 1). In the case of the instrumental implementation of the polarimetric system shown in Figure 1, this parameter is related to the wavelength λ of readout radiation generated by the laser (item 1), focal length F of the Fourier-transforming lens (item 5), and values of the pixel sizes $\Delta x_{p},\Delta x_{d}$ of the gene-based spatial light modulator (item 4) and detector in plane 7. The characteristic speckle size (in the case of random distributions of ${\left(\Delta \varphi_{ij}\right)}_{2n\times 2n}^{x}$ and ${\left(\Delta \varphi_{ij}\right)}_{2n\times 2n}^{y}$) or the size of diffraction peaks (in the case of uniform distributions of ${\left(\Delta \varphi_{ij}\right)}_{2n\times 2n}^{x}$ and ${\left(\Delta \varphi_{ij}\right)}_{2n\times 2n}^{y}$) in plane 7 is determined by the ratio $\lambda F/N\Delta x_{p}$. On the other hand, a characteristic size of the formed diffraction pattern in the plane 7 relates to the ratio $\lambda F/\Delta x_{p}$. An increase in F or λ leads to an increase in transverse dimensions of the diffraction pattern in plane 7 and, thereby, to an increase in its acquired details at fixed values of the number and size of detector pixels. In the case of computer simulation, a decrease in $K_{sc}$ leads to an increase in the details of the synthesized diffraction pattern and corresponding distributions of the local values of the Stokes vector in the paraxial region (similarly to a decrease in F or λ in the case of instrumental implementation).

**Figure 2:**
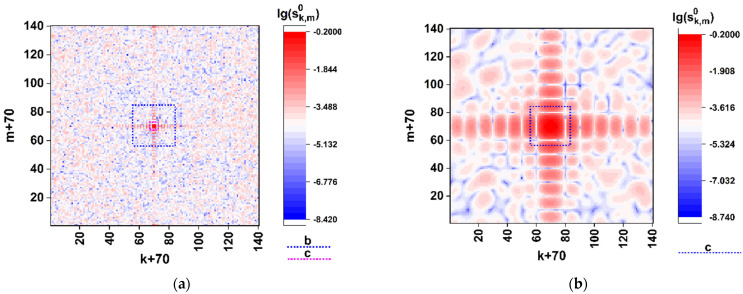

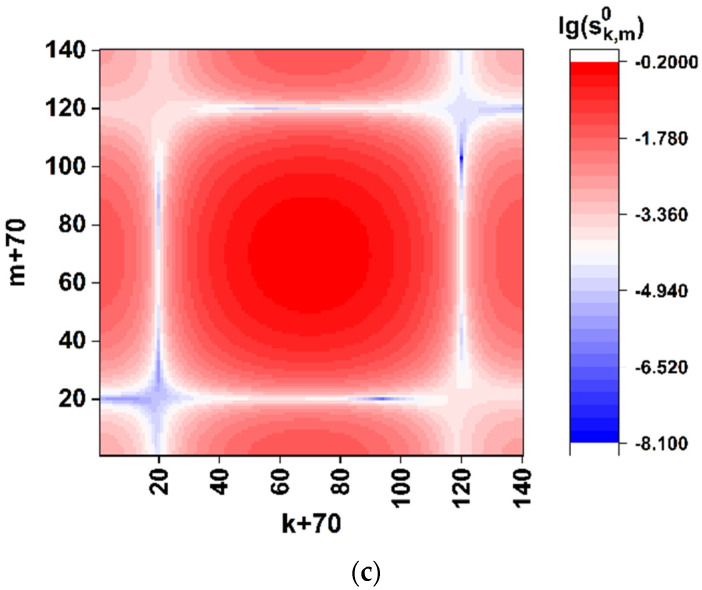
Effect of detailing the logarithmic intensity distribution $lg(s_{k,m}^{0})$ of the diffracted light in plane 7 (Figure 1) with a decrease in the scale factor $K_{sc}$ (the results of computer simulation for the model spike gene of the SARS-CoV-2 Wuhan strain). (**a**) $K_{sc}$ = 0.5; (**b**) $K_{sc}$ = 0.1; (**c**) $K_{sc}$ = 0.02.

As an example, Figure 2 illustrates the effect of detailing with the decreasing $K_{sc}$ for the synthesized model distribution $s_{k,m}^{0}$ in case of application of the above-described algorithm (Equations (3) and (4)) to the model spike gene of the SARS-CoV-2 virus [33] Wuhan strain (the GenBank Acc. No. EPI_ISL_402124) [34]. The 1225-element fragment of the corresponding triplet sequence taken beginning from the start codon was transformed to the (70 × 70)-element quasi-random phase screen using the above-described coding procedure. The modulation parameters were established as $K_{\varphi }^{x}\left(i,j\right)=K_{\varphi }^{y}\left(i,j\right)=0$ and $f\left(i,j\right)=\left(\pi /2\right)\cdot {\left(a_{ij}\right)}_{2n\times 2n}$. The dotted squares on the panels (a) and (b) highlight fragments of the synthesized distribution, which are expanded over the entire analyzed area with a decrease in $K_{sc}$.

The source codes for converting (A,C,T,G) sequences into phase-modulating matrices ${\left(a_{ij}\right)}_{2n\times 2n}$ (MatLab software environment) and for synthesizing the $s_{k,m}^{0÷3}$ distributions in the diffraction plane (C programming language) are presented in the Supplementary Materials of this article with the necessary comments.

According to the basic principles of polarization optics [31], the first component of the Stokes vector $s_{k,m}^{0}$ determines the total intensity of the diffracted field in the k,m point. The second component $s_{k,m}^{1}$ characterizes the normalized difference of intensities of x- and y- linearly polarized components in the same point. The third component $s_{k,m}^{2}$ relates to a similar difference in the $\left(x^{\prime },y^{\prime }\right)$ coordinate system rotated at the angle of π/4 with respect to the basic coordinates (x,y). Finally, the $s_{k,m}^{3}$ component characterizes a contribution of a circular polarization in the local polarization state of the diffraction field. The value $s_{k,m}^{3}=1$ corresponds to the pure right circular polarization of the diffracted light at the point. Accordingly, $s_{k,m}^{3}=-1$ indicates the left circular polarization.

**Figure 3:**
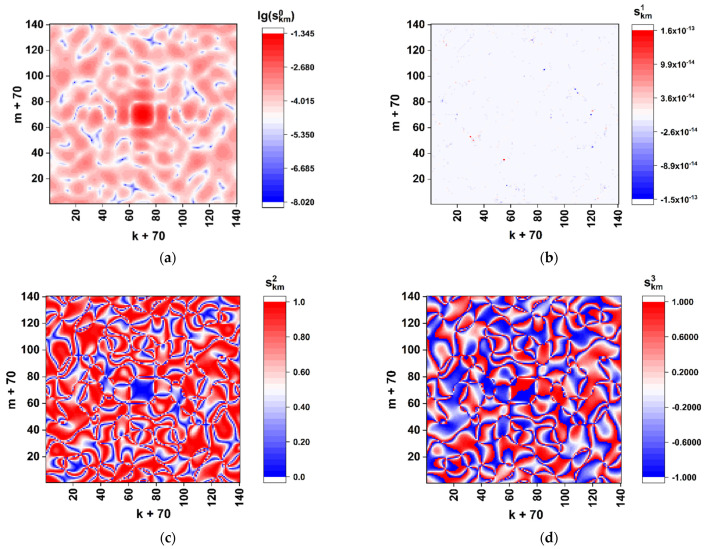
Color maps of the local polarization states in the modeled diffraction pattern for the nucleotide sequence of the model spike gene of the SARS-CoV-2 Wuhan strain. The scale factor $K_{sc}$ is equal to 0.1. (**a**) $\mathrm{lg}\left(s_{k,m}^{0}\right)$; (**b**) $s_{k,m}^{1}$; (**c**) $s_{k,m}^{2}$; (**d**) $s_{k,m}^{3}$.

As an example, consider distributions of the $s_{k,m}^{0÷3}$ values in the (k,m) plane in case of the SARS-CoV-2 Wuhan strain [34]. Figure 3 displays the color maps of $s_{k,m}^{0÷3}$ distributions recovered using the Formulas (4)–(6) in case of $K_{\varphi }=\pi$ and $K_{r}=\pi /2$. The scale factor $K_{sc}$ was set equal to 0.1 in order to refine the features of distributions in the paraxial zone. Note that the used phase modulation algorithm leads to a two-grade (binary) polarization structure of the boundary field (directly behind the synthesized phase screen). This follows from the identity of the x-component states of the boundary field in the regions behind the elements $a_{ij}$ equal to 0 and 2 (the principal value of the phase delay of the readout beam equal to 0) and equal to 1 and 3 (the principal value of the phase delay equal to π). With the uniform phase delay $K_{r}=\pi /2$ of the y-component to the x-component (see Formula (2)), the model boundary field distribution will be fragmented to a set of equal-sized fragments with the right circular or left circular polarization.

The obtained distribution of the normalized total intensity $s_{k,m}^{0}$ (Figure 3a) exhibits an expressed speckle modulation caused by a random distribution of the $\left(a_{ij}\right)$ matrix elements. At the same time, the on-axis total intensity $s_{0,0}^{0}$ has a sufficiently non-zero value; this feature indicates significantly different frequencies of occurrence of nucleotides during their random selection from the analyzed sequence.

In particular, the frequency analysis of the nucleotide sequence of the spike gene SARS-CoV-2 Wuhan strain gives the following relative weights of various nucleotides in the sequence: A– ≈ 0.2955; C– ≈ 0.1894; T– ≈ 0.3333; G– ≈ 0.1818. In the case of equal relative weights, the component $s_{0,0}^{0}$ is expected to fall to zero. The remarkable features are close-to-zero values of the $s_{k,m}^{1}$ component and symmetry properties of the $s_{k,m}^{2}$ and $s_{k,m}^{3}$ distributions: the axial symmetry of the first distribution ($s_{k,m}^{2}=s_{140-k,140-m}^{2}$) and the antisymmetric character of $s_{k,m}^{3}$ ($s_{k,m}^{3}=s_{140-k,140-m}^{3}$). These features result from the applied phase-modulation algorithm, when only left-circular or right-circular local polarization states of the transmitted light field occur behind the phase screen.

### 2.3. Binary Mapping of Extreme Local Polarization States

A qualitative analysis of the relationship between the features of the analyzed target gene of the SARS-CoV-2 Wuhan strain structure and $s_{k,m}^{0÷3}$ distributions in the synthesized diffraction pattern allows us to suggest that positions of $s_{k,m}^{3}$ extreme values, which are close to 1 or −1, are very sensitive to the structural changes in the analyzed (A,C,T,G) sequence. This follows from rather stringent formation conditions for such states in the diffracted field: the equality of the amplitudes of the x- and y-components of the diffracted field arriving at a given point; the proximity of the phase shift between them to π/2 or 3π/2.

Accordingly, the following procedure for mapping close-to-extreme local polarization states can be considered:

(7) $$\left\{ \begin{array}{l} s_{k,m}^{p}>\left(<\right)s_{th}^{p}\to {\tilde{s}}_{k,m}^{p}=1;\\ s_{k,m}^{p}<\left(>\right)s_{th}^{p}\to {\tilde{s}}_{k,m}^{p}=0, \end{array}\right.$$

where p=1÷3, $s_{th}^{p}$ defines the discrimination level for a chosen component of the Stokes vector and ${\tilde{s}}_{k,m}^{p}$ is the binarized value of the chosen component. Designations >(<) and <(>) used in (7) are due to the fact that the values of $s_{k,m}^{p}$ can vary from −1 to 1. Accordingly, when choosing positive extreme values, “>” is used in the first line and “<” in the second line of (7). In the opposite case of negative extreme values, the order of “<” and “>” is reversed.

**Figure 4:**
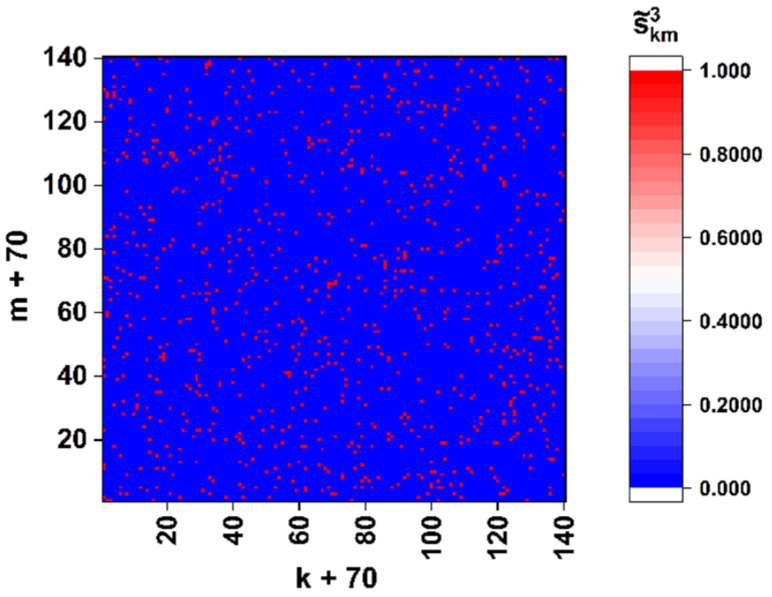
Binary distribution ${\tilde{s}}_{k,m}^{3}$ for the nucleotide sequence of the model spike gene of the SARS-CoV-2 Wuhan strain. The discrimination threshold $s_{th}^{3}$ is −0.99 and the scale factor is 0.5.

As an example, Figure 4 displays the “panoramic” ($K_{sc}=$ 0.5) binary map of the close-to-left-circular local polarization states ($s_{th}^{3}=$−0.99) in the diffraction pattern corresponding to the sequence of the spike gene of the SARS-CoV-2 Wuhan strain (see Figure 3d). We can introduce the density of extreme states as a parameter dependent on the applied discrimination threshold $\Omega_{th}^{p}=N_{th}^{p}/4N^{2}$, where $N_{th}^{p}$ is the total number of points with ${\tilde{s}}_{k,m}^{p}=1$ within the analyzed diffraction pattern.

In particular, analysis of the model binary maps ${\tilde{s}}_{k,m}^{3}$ for the target gene of the Wuhan strain shows that the density of close-to-left-circular polarization states falls as $\Omega_{th}^{3}\propto {\left(1+s_{th}^{3}\right)}^{0.5}$ with $s_{th}^{3}\to$ −1.

Diversity in the nucleotide sequences corresponding to the model nucleotide sequences of three different variants of the SARS-CoV-2 spike gene can be quantified by evaluation of the correlation coefficient between the corresponding binary maps:

(8) $$R_{1,2}^{p}=\frac{\sum_{k,m}{\tilde{s}}_{k,m}^{1,p}{\tilde{s}}_{k,m}^{2,p}}{\sum_{k,m}{\left({\tilde{s}}_{k,m}^{1,p}\right)}^{2}}$$

where index “1” corresponds to the “basic” sequence of nucleotides and “2” defines the sequence for which the diversity is quantified.

### 2.4. The Choice of Nucleotide Sequences for Modeling

A numerical experiment to verify the approach under consideration was carried out for three nucleotide sequences of the spike gene derived from the model strains. The most common SARS-CoV-2 variants such as the Wuhan [34], Delta [35], and Omicron [36] were examined. Figure S1 in the Supplementary Materials displays the numbers of mismatches in the standard alignment of the corresponding spike nucleotide and amino acid sequences. The homology level between the sequences of either Delta or Omicron variants compared to the Wuhan reference sequence is relatively high (≥99%). In particular, a pairwise sequence alignment using the EMBOSS Needle on-line tool (https://www.ebi.ac.uk/Tools/psa/emboss_needle/ (accessed on 16 October 2022)) gives the identity value for the “Delta–Wuhan” pair as 99.6% (3807/3822). The similar value for the “Omicron–Wuhan” pair is 99.0% (3783/3822). The number of unrecognized nucleotides (gaps) is six in case of the “Delta” sequence and nine for the “Omicron” sequence. Accordingly, the number of identified mismatches is 9 for the “Delta–Wuhan” pair and 30 for the “Omicron–Wuhan” pair. Table S1 in the Supplementary Materials presents the differences in the nucleotide triplet sequences and the corresponding amino acid substitutions.

In the course of the ${\left(a_{ij}\right)}_{2n\times 2n}$ matrices synthesis, the fragments of (A,C,T,G) sequences with the length of 1225 triples (3675 nucleotides) were selected for each analyzed strain, beginning from the start codons. Accordingly, the sizes of the synthesized phase-modulating matrices ${\left(a_{ij}\right)}_{2n\times 2n}$ were 70 × 70. During the synthesis, the gaps in the “Delta” and “Omicron” sequences were filled by the corresponding nucleotides taken from the “Wuhan” sequence. Note that, despite the shorter length of the selected fragments compared to the original sequences (3675 against 3822), all mismatched nucleotides (and, accordingly, triplets) were in the selected fragments.

## 3. Results

### 3.1. Gene Structure Diversity and Correlation of the Binary Maps of Local Polarization States

Sensitivity of the introduced correlation coefficient $R_{1,2}^{p}$ (Equation (8)) to the local changes in the structure of the initial nucleotide reference sequence was analyzed by gradual replacement of individual nucleotides in the triplets (for example, A→C, T→G, etc.). Due to quasi-stochastic distributions of the elements of the generated matrices ${\left(a_{ij}\right)}_{2n\times 2n}$, it is necessary to average the coefficient $R_{1,2}^{p}$ over a sample set of the binary distributions ${\tilde{s}}_{k,m}^{2,p}$. This sample set was generated as a result of random changes of a given number of the nucleotides $N_{s}$ in the original sequence. The nucleotide sequence of the spike gene derived from the SARS-CoV-2 Wuhan variant was considered as the initial sequence with the corresponding binary distribution ${\tilde{s}}_{k,m}^{1,p}$. The sample size used for averaging was equal to 10. The number $N_{s}$ of replaced nucleotides varied from 0 to 30. In the modeling, the extreme states of the third ($s_{k,m}^{2}$) and fourth ($s_{k,m}^{3}$) components of the Stokes vector were considered; accordingly, discrimination thresholds were chosen as $s_{th}^{2}=$ 0.99 and $s_{th}^{3}=$−0.99.

**Figure 5:**
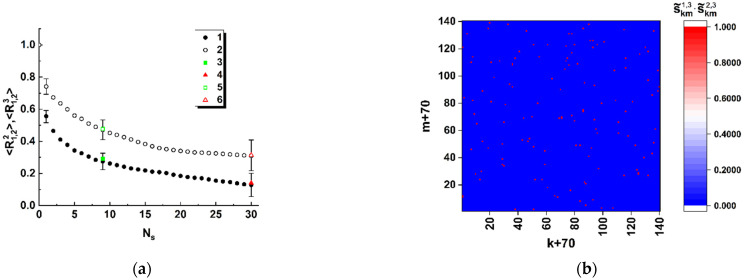
(**a**) Correlation coefficients of the binary maps of the third and fourth components of the Stokes vector against the number of substitutions in the reference sequence of the nucleotides. The sequence of the SARS-CoV-2 spike gene for the model Wuhan variant is applied as a reference item. Associations: 1, 3, 4—the fourth component of the Stokes vector; 2, 5, 6—the third component. 1, 2–pairs “reference/changed sequence”; 3, 5—“Wuhan/Delta” correlation; 4, 6—“Wuhan/Omicron” correlation. (**b**) Superposition of the binary maps $\left({\tilde{s}}_{k,m}^{3}\right)$ for the Wuhan and Omicron variants. The scale factor $K_{sc}$ is equal to 0.5.

The results of modeling are presented in Figure 5, a as the values of $\langle R_{1,2}^{2}\rangle$ and $\langle R_{1,2}^{3}\rangle$ against the number $N_{s}$ of nucleotide substitutions. As an example of decorrelation of the binary maps due to changes in the sequence structure, Figure 5b displays an effect of superposition $\left({\tilde{s}}_{k,m}^{1,3}{\tilde{s}}_{k,m}^{2,3}\right)$ of the binary maps corresponding to the Wuhan and Omicron variants (compare the densities of the unit states in Figure 4 and Figure 5b). We can see that the substitution of only a few nucleotides in the analyzed sequence with respect to the reference leads to a sharp decrease in the number of coinciding points on the binary maps corresponding to the analyzed and reference sequences.

In Figure 5a, selectively shown error bars correspond to the confidence level of 0.9; empty and filled square and triangle markers display the coefficients $R_{1,2}^{2}$, $R_{1,2}^{3}$ of the binary map correlation for the pairs Wuhan–Delta and Wuhan–Omicron model variants.

### 3.2. Frequency Counting of Nucleotide Sequences Using the Polarization Encoding

The polarization encoding potential is not limited only to quantification of differences between nucleotide sequences for the analyzed and “basic” or reference variants of the same biological object. Let us consider a technique for the frequency analysis of nucleotide sequences, which is also based on the principle of polarization encoding. The relative frequencies of occurrence of the basic nucleotides A,C,T,G in the analyzed sequence of triplets can be described by the (4×4) matrix of the following form:

(9) $$\left(\begin{array}{cccc} \rho_{0}^{A} & \rho_{1}^{A} & \rho_{2}^{A} & \rho_{3}^{A}\\ \rho_{0}^{C} & \rho_{1}^{C} & \rho_{2}^{C} & \rho_{3}^{C}\\ \rho_{0}^{T} & \rho_{1}^{T} & \rho_{2}^{T} & \rho_{3}^{T}\\ \rho_{0}^{G} & \rho_{1}^{G} & \rho_{2}^{G} & \rho_{3}^{G} \end{array}\right)$$

where $\rho_{0÷3}^{A÷G}=N_{0÷3}^{A÷G}/N^{2}$ and $N_{0÷3}^{A÷G}$ are the amounts of triplets containing 0÷3 nucleotides of the corresponding type (A÷G). The matrix (9) can be interpreted as the density matrix of the triplet states. It is obvious that the following normalization condition takes place for the row elements: $\sum_{A,C,T,G}\rho_{0÷3}^{A÷G}=1$. Additionally, another normalization condition is valid for the introduced density matrix of the triplet states: $\left(1/3\right)\sum_{A,C,T,G}\sum_{s=1}^{3}\rho_{s}^{A÷G}=1$.

For example, the density matrix of the triplet states for the sequence of the reference Wuhan variant obtained by a direct computer analysis of the triplet sequence is:

(10) $${\left(\rho_{0÷3}^{A÷G}\right)}_{W}\approx \left(\begin{array}{cccc} 0.3861 & 0.3698 & 0.2155 & 0.0286\\ 0.5159 & 0.4033 & 0.0776 & 0.0032\\ 0.3298 & 0.3878 & 0.2351 & 0.0473\\ 0.5624 & 0.3322 & 0.1029 & 0.0025 \end{array}\right)$$

Next, consider the following two-stage algorithm for transforming the initial matrix ${\left(a_{ij}\right)}_{2n\times 2n}$ into the matrices of the phase shifts ${\left(\Delta \varphi_{ij}\right)}_{2n\times 2n}^{x}$ and ${\left(\Delta \varphi_{ij}\right)}_{2n\times 2n}^{y}$ (see Equation (3)):

(11) $$\left\{ \begin{array}{l} K_{\varphi }^{x}\left(i,j\right)=\left\{ \begin{array}{l} \pi /2\;\left(first\;stage\right)\\ \pi /3\;\;(second\;stage) \end{array}\right.,\left(i,j\right)=2s+1;\;s=0,1,2,.....,N-1;\\ K_{\varphi }^{x}\left(i,j\right)=0\;in\;all\;other\;cases;\\ K_{\varphi }^{y}\left(i,j\right)=0;\\ f\left(i,j\right)=0. \end{array}\right.\;$$

Note that, in contrast to the transformation algorithm used in Section 2.2, we assume different values of the phase modulation factor $K_{\varphi }$ for the x- and y-components. In addition, there is not any phase modulation or retardation for the y-component, which is considered as a reference component. The suggested transformation rule, in combination with the used encoding algorithm, provides selection of the ${\left(a_{ij}\right)}_{2n\times 2n}$ matrix elements associated with adenine (A). Other C,T,G-associated matrix elements do not specifically affect the distribution of the diffracted readout field. Changing the selection rule, we can choose any other basic nucleotides (C, T, or G) as a target object.

Consider the normalized on-axis amplitudes of the x, y-components $E_{0,0}^{x},\;E_{0,0}^{y}$ of the diffracted field (see Equation (5)) for the suggested transformation rule. It can be shown that at the first stage the normalized x, y-components $E_{0,0}^{x},\;E_{0,0}^{y}$ are expressed as:

(12) $$\left\{ \begin{array}{l} E_{0,0}^{x}=1-\frac{1}{4}[\left(\rho_{1}^{A}+\rho_{2}^{A}+\rho_{3}^{A}\right)+\rho_{1}^{A}\cdot \exp\left(\tilde{j}\frac{\pi }{2}\right)+\\ +\rho_{2}^{A}\cdot \exp\left(\tilde{j}\pi \right)+\rho_{3}^{A}\cdot \exp\left(\tilde{j}\frac{3\pi }{2}\right)];\\ E_{0,0}^{y}=1. \end{array}\right.$$

The multiplication factor (1/4) occurs due to the relationship between the number of submatrices corresponding to the triplets in the nucleotide sequence and the number of elements in the matrix ${\left(a_{ij}\right)}_{2n\times 2n}$. Accordingly, the real and imaginary components of the x, y-components $E_{0,0}^{x},\;E_{0,0}^{y}$ are:

(13) {Re(E0,0x)=1−0.25⋅ρ1A−0.5⋅ρ2A−0.25⋅ρ3A;Im(E0,0x)=0.25⋅(ρ1A−ρ3A);Re(E0,0y)=1;Im(E0,0y)=0.

At the second stage of the readout procedure, the normalized on-axis components of the diffracted light field are:

(14) $$\left\{ \begin{array}{l} E_{0,0}^{x}=1-\frac{1}{4}[\left(\rho_{1}^{A}+\rho_{2}^{A}+\rho_{3}^{A}\right)+\rho_{1}^{A}\cdot \exp\left(\tilde{j}\frac{\pi }{3}\right)+\\ +\rho_{2}^{A}\cdot \exp\left(\tilde{j}\frac{2\pi }{3}\right)+\rho_{3}^{A}\cdot \exp\left(\tilde{j}\pi \right)];\\ E_{0,0}^{y}=1, \end{array}\right.$$

and

(15) {Re(E0,0x)=1−0.125⋅ρ1A−0.375⋅ρ2A−0.5⋅ρ3A;Im(E0,0x)=(3/8)⋅(ρ1A+ρ2A);Re(E0,0y)=1;Im(E0,0y)=0.

Considering definitions of the third and fourth components of the local Stokes vector $s_{k,m}^{2}$ and $s_{k,m}^{3}$ (Equation (6)), we can expect that, under the conditions $K_{\varphi }^{y}\left(i,j\right)=0$ and f(i,j)=0, the terms $\left|E_{0,0}^{x}\right|\left|E_{0,0}^{y}\right|\cos\left(\delta_{0,0}\right)$ and $\left|E_{0,0}^{x}\right|\left|E_{0,0}^{y}\right|\sin\left(\delta_{0,0}\right)$ are reduced to the following forms:

(16) {|E0,0x||E0,0y|cos(δ0,0)=Re(E0,0x);|E0,0x||E0,0y|sin(δ0,0)=Im(E0,0x).

Thus, taking into consideration Equations (6), (13), (15) and (16), we arrive to the system of linear equations for $\rho_{0÷3}^{A}$, where the upper indices I and II correspond to the above considered modulation readout stages:

(17) $$\left\{ \begin{array}{l} 0.25\cdot \left(\rho_{1}^{A}-\rho_{3}^{A}\right)={\left(s_{0,0}^{0}\cdot s_{0,0}^{3}\right)}^{I};\\ 1-0.25\cdot \rho_{1}^{A}-0.5\cdot \rho_{2}^{A}-0.25\cdot \rho_{3}^{A}={\left(s_{0,0}^{0}\cdot s_{0,0}^{2}\right)}^{I};\\ 1-0.125\cdot \rho_{1}^{A}-0.375\cdot \rho_{2}^{A}-0.5\cdot \rho_{3}^{A}={\left(s_{0,0}^{0}\cdot s_{0,0}^{2}\right)}^{II}. \end{array}\right.$$

The model on-axis values $s_{0,0}^{0},\;s_{0,0}^{2},\;s_{0,0}^{3}$ for the reference Wuhan variant for the considered encoding-readout procedure are: ${\left(s_{0,0}^{0}\right)}^{I}\approx$ 0.8178, ${\left(s_{0,0}^{2}\right)}^{I}\approx$ 0.9693, ${\left(s_{0,0}^{3}\right)}^{I}\approx$ 0.1043, ${\left(s_{0,0}^{0}\right)}^{II}\approx$ 0.8767, ${\left(s_{0,0}^{2}\right)}^{II}\approx$ 0.9795, and ${\left(s_{0,0}^{3}\right)}^{II}\approx$ 0.1446. Accordingly, the solution to the system (17) with the defined free terms is: $\rho_{1}^{A}\approx$ 0.3697, $\rho_{2}^{A}\approx$ 0.2155, and $\rho_{3}^{A}\approx$ 0.0285 (compared with the results of direct computation (10)).

### 3.3. Only-Retardation Polarization Encoding

**Figure 6:**
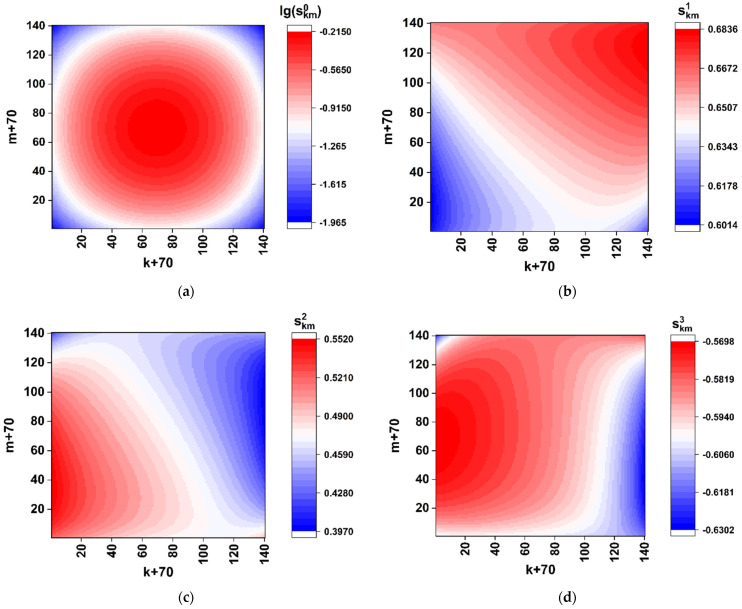
Color maps of local polarization states in the paraxial region of the modeled diffraction pattern for the nucleotide sequence of the spike gene of the SARS-CoV-2 Wuhan variant. The case is of only-retardation modulation. The scale factor $K_{sc}$ is equal to 0.01. (**a**) $\mathrm{lg}\left(s_{k,m}^{0}\right)$; (**b**) $s_{k,m}^{1}$; (**c**) $s_{k,m}^{2}$; (**d**) $s_{k,m}^{3}$.

The considered algorithm (3) of phase modulation of the x- and y-components in the readout beam is based on the possibility of setting independent values of the phase shift ($K_{\varphi }^{x,y}\left(i,j\right)$) and retardation ($f\left\{K_{\varphi }{\left(a_{ij}\right)}_{2n\times 2n}\right\}$) for each element of the synthesized matrix (${\left(a_{ij}\right)}_{2n\times 2n}$). From the point of view of instrumental implementation of polarization encoding of the gene-based (GB) synthesized matrices, this procedure can be carried out using cascaded liquid-crystal-based spatial light modulators (LC SLMs). In the case of application of a single SLM unit without any cascading, only retardation (phase shift between the x- and y-components) can be set independently for each SLM element with the same values of $K_{\varphi }^{x,y}\left(i,j\right)$ across the SLM aperture. Accordingly, the corresponding modulation algorithm can be considered in the following form:

(18) $$\begin{array}{l} {\left(\Delta \varphi_{ij}\right)}_{2n\times 2n}^{x}=0;\\ {\left(\Delta \varphi_{ij}\right)}_{2n\times 2n}^{y}=f\left\{{\left(a_{ij}\right)}_{2n\times 2n}\right\}, \end{array}$$

with a simple multiplication rule of retardation modulation:

(19) $$f\left\{{\left(a_{ij}\right)}_{2n\times 2n}\right\}=K_{r}\cdot {\left(a_{ij}\right)}_{2n\times 2n},$$

where $K_{r}$ is the modulation factor of the phase retardation. Note that equal values of the phase shift for all the SLM elements cause remarkable changes in the formed diffraction pattern compared to the above considered case of simultaneous modulation of the phase shift and retardation (Figure 2 and Figure 3). These features are illustrated by the near-axis $\left(s_{k,m}^{0}÷s_{k,m}^{3}\right)$ distributions for the GB matrix ${\left(a_{ij}\right)}_{2n\times 2n}$ associated with a nucleotide sequence of the reference Wuhan variant (Figure 6). The phase retardation factor is equal to π/2 (i.e., the modulation rule corresponds to that used for the first-stage procedure in Section 3.2). The distribution of the normalized total intensity $\left(s_{k,m}^{0}÷s_{k,m}^{3}\right)$ corresponds to the classical diffraction pattern from a square aperture with the local random deviations caused by phase modulation of the y-component (Equations (18) and (19)). Note that, because of small values of the diffracted light intensity outside the near-axis zone (the zero-order diffraction maximum), robust evaluations of the normalized local values $\left(s_{k,m}^{0}÷s_{k,m}^{3}\right)$ outside the paraxial zone are instrumentally difficult to implement. Accordingly, the previously considered algorithm for discrimination of local polarization states and subsequent binary mapping of discriminated states should be modified based on the analysis of distributions in the axial zone (under condition of small values of the $K_{sc}$ parameter) and the absence of local values of the $s_{k,m}^{2}$, $s_{k,m}^{3}$ components close to extreme magnitudes (±1, see Figure 5). As one of the possible examples of such a modified approach to the analysis of local polarization states, consider the following algorithm for binary mapping of the paraxial zone:

(20) $$\left\{ \begin{array}{l} \left(\left(s_{k,m}^{1}<s_{th}^{1}+\Delta s_{th}^{1}\right)\&\left(s_{k,m}^{1}>s_{th}^{1}-\Delta s_{th}^{1}\right)\right)\&\\ \&\left(\left(s_{k,m}^{2}<s_{th}^{2}+\Delta s_{th}^{2}\right)\&\left(s_{k,m}^{2}>s_{th}^{2}-\Delta s_{th}^{2}\right)\right)\to {\tilde{s}}_{k,m}=1;\\ else\to {\tilde{s}}_{k,m}=0. \end{array}\right.$$

Note that this is one of the possible algorithms for discrimination of local polarization states for recovery of the binary map $\left({\tilde{s}}_{k,m}\right)$ associated with the given reference sequence of nucleotides. The threshold values $s_{th}^{1}$ and $s_{th}^{2}$ and maximum deviations $\Delta s_{th}^{1}$ and $\Delta s_{th}^{2}$ are selected based on the intervals of the changes in the corresponding components of the Stokes vector in the axial zone. Another factor that determines the choice is the required sensitivity of obtained binary maps to variability of the analyzed sequences.

**Figure 7:**
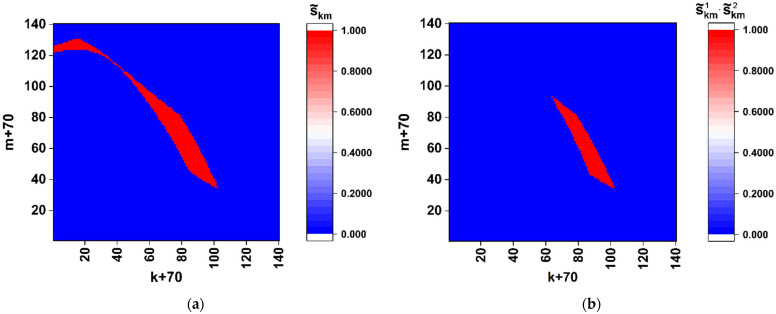
(**a**) The binary map of the near-axis local polarization states selected using the rule (20) for the nucleotide sequence of the spike gene of the SARS-CoV-2 Wuhan variant; (**b**) the result of superposition of the near-axis binary maps for the Wuhan variant and the relevant nucleotide sequence of the spike gene of the SARS-CoV-2 Omicron variant. The scale factor $K_{sc}$ is equal to 0.01.

As an example, Figure 7a displays the corresponding binary map $\left({\tilde{s}}_{k,m}\right)$ for the GB retardation-modulating matrix corresponding to the Wuhan variant. Panel 7b represents the result of superposition of the binary maps for the Wuhan and Omicron variants. In accordance with the modeled datasets presented in Figure 6, the threshold and maximum deviations were set equal to: $s_{th}^{1}=$ 0.655, $s_{th}^{2}=$ 0.475, $\Delta s_{th}^{1}=$0.005, and $\Delta s_{th}^{1}=$ 0.005. The correlation coefficient $R_{1,2}=\sum_{k,m}{\tilde{s}}_{k,m}^{1}{\tilde{s}}_{k,m}^{2}/\sum_{k,m}{\left({\tilde{s}}_{k,m}^{1}\right)}^{2}$ (the upper index “1” corresponds to the Wuhan variant and “2” corresponds to the Omicron variant) is equal to ≈0.536. Note that the discrimination condition (20) also defines a set of coordinates k,m; for those, the component $s_{k,m}^{3}$ varies within a certain range (it follows from the normalization condition ${\left(s_{k,m}^{1}\right)}^{2}+{\left(s_{k,m}^{2}\right)}^{2}+{\left(s_{k,m}^{3}\right)}^{2}=1$).

**Figure 8:**
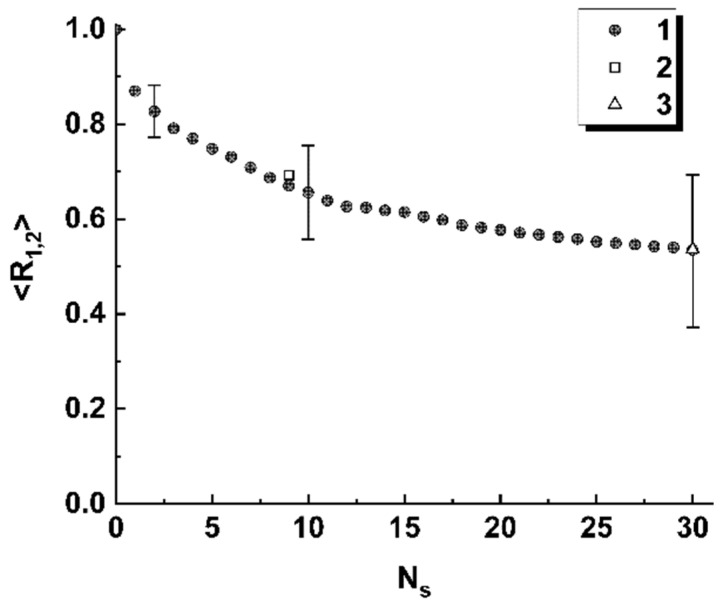
Model values of the correlation coefficient $\langle R_{1,2}\rangle$ against the number of substitutions (1) and correlation coefficients for the nucleotide sequence of the “Wuhan–Delta” (2) and “Wuhan–Omicron” (3) pairs. The case of the near-axis readout of the local polarization states and application of the discrimination rule (20) is considered.

Figure 8 displays the results of modeling the correlation $\langle R_{1,2}\rangle$ between the reference (the reference Wuhan variant) and changed-by-substitutions binary maps for the considered case of the near-axis readout of the local polarization states. The empty square and triangle correspond to the correlation coefficients for the “Wuhan–Delta” and “Wuhan–Omicron” pairs. Selectively shown error bars correspond to the confidence level of 0.9.

## 4. Discussion

Thus, the obtained model data allow us to conclude that spatial distributions of the discriminated local polarization states in the output plane of the considered polarimetric system (Figure 1) are adequately sensitive to the local changes in the structure of the sequences of basic nucleotides. Analyzing the behavior of the introduced coefficients of the correlation between binarized distributions (Figure 5a and Figure 8), we can note that the considered approach is characterized by maximum sensitivity to the diversity in the structure of a pair of sequences when the number of differing nucleotides is small (from 1 to 3). The general trend in the behavior of obtained model dependences is a significant increase in the variance of coefficients of the correlation between the reference and analyzed binary distributions as the number of differences increases. Accordingly, the discussed polarimetric technique is ineffective in terms of estimating the number of changed nucleotides in the analyzed sequence with respect to the reference sequence at large values of the changed nucleotides. However, its efficiency is acceptable for small differences in the structure of sequences. This leads to the key difference between the discussed method of polarization encoding and the previously discussed method for the synthesis of GB speckles [28], where opposite tendencies occur (low sensitivity to small changes in the structure of nucleotide sequences and its increase with an increasing number of differences).

As expected at the beginning of the study, spatial distributions of extreme local values of the fourth component of the Stokes vector, which are close to 1 or −1, are characterized by maximal sensitivity to small local changes in nucleotide sequences (see set 1 of the model data in Figure 5a). Accordingly, in the zones of diffraction plane 7 (Figure 1), which correspond to extreme states of the fourth component of the Stokes vector, the second and third components have values very close to 0. This feature can also be used for recovery of a binary map, which is actually a unique identifier of the analyzed nucleotide sequence. In this case, the unit values are attributed to those zones for which the local values of the second and third components simultaneously take on the values less than the specified small thresholds. The best condition for reading out panoramic distributions of extreme local values of the fourth component of the Stokes vector, similar to that shown in Figure 4 is suppression of the paraxial diffraction maximum in readout plane 7 (Figure 1). On the other hand, complete suppression of this maximum in the case of applied algorithms of phase modulation in the readout beam is unattainable due to different relative weights of the base nucleotides (A,C,T,G) in the analyzed sequences.

Among the considered model cases of polarization encoding-readout of nucleotide sequences, the case of only-retardation modulation and near-axis readout of local polarization states is characterized by minimal sensitivity to small changes in the structure of the sequences (compare Figure 5a and Figure 8). At the same time, this encoding-readout technique makes it possible to carry out a frequency analysis of nucleotide sequences using the principle of selecting a specific nucleotide at the stage of synthesizing the phase-modulating matrix.

Note that a pilot computer simulation of binary mapping of extreme polarization states in gene-based diffraction patterns was previously carried out [37] for the target gene p72 of three different strains of the model African swine fever virus (ASF). The fragments of (A,C,T,G) sequences with the length of 625 triplets for three different strains (HuB20 (NCBI GenBank access number: MW521382. 1, [30]), Zaire (NCBI GenBank access number MW296952.1, [38]), and Ulyanovsk 19/WB-5699 (NCBI GenBank access number MW306192.1, [39]) were converted into the phase modulating matrices ${\left(a_{ij}\right)}_{2n\times 2n}$ in accordance with the above-described rule (Equations (1) and (2)). The results of estimations of the correlation coefficients $R_{1,2}^{p}$ for the pairs “HuB20-Ulyanovsk 19/WB-5699” and “HuB20- Zaire “ (the sequence fragment for HuB20 was considered as a reference item) also demonstrated significant changes in $R_{1,2}^{p}$ upon substitution of small numbers of nucleotides $N_{s}$ and saturation of the sensitivity of $R_{1,2}^{p}$ to $N_{s}$ with an increase in the number of substitutions.

As an example of possible instrumental implementation of the discussed approach, let us consider the design of a polarimetric analyzer of A,C,T,G sequences (Figure 1), which is based on commercially available optical units. For example, the transmissive liquid crystal spatial light modulator (LC SLM) of the LS2012 type from the Holoeye Photonics AG (Germany) (or other available LC SLM unit with similar characteristics) can be used as a gene-based phase modulator (item 4 in Figure 1). Parameters of the LS2012 unit (1024 × 768 resolution, maximum phase modulation depth of 1.8π at the readout wavelength of 532 nm, input frame rate of 60 Hz, and 8-bit pixel addressing) in combination with supporting software will allow for implementation of the discussed algorithms of transcoding A,C,T,G sequences into two-dimensional phase matrices. A general purpose continuous-wave laser (e.g., a DPSS or He-Ne unit) can be applied as a source of readout radiation (item 1 in Figure 1); in particular, the DJ532-40 (with the wavelength of 532 nm) or HNL100LB (with the wavelength of 633 nm) laser from the Thorlabs Inc. (Newton, NJ, USA) will be acceptable as a source of readout light. To acquire local polarization states of the diffracted laser light in the output plane of the polarimetric system (item 7 in Figure 1), the Kiralux polarization camera CS505MUP1 with the 5-megapixel monochrome CMOS sensor (the product of the Thorlabs Inc., Newton, NJ, USA) can be used in combination with an appropriate objective lens. Note that instrumental implementation of the polarimetric system for analyzing the (A,C,T,G) sequences is not limited to the set of considered equipment and the configuration shown in Figure 1. In particular, it can be carried out using the principle of cascading of SLM units by their sequential arrangement with the matching optical elements. This can significantly expand the functionality of the polarimetric method for identifying and analyzing the differences in the genetic structure. Creation of an instrumental prototype of the considered polarimetric system and its thorough experimental verification using a wide set of genetic data in the form of (A,C,T,G) sequences for various strains of biological objects is the subject of our further work.

It should be noted that the functionality of the discussed approach is far from being limited to the considered particular cases; for example, we can consider the encoding-readout schemes, which allow for identification of the specific nucleotides’ positions and their combinations in the analyzed sequences. These points are the object of further research together with the issues of instrumental implementation of the method.

## Data Availability

All analyzed nucleotide sequences were taken from open sources cited in the reference list. The source codes for the developed software are presented in the Supplementary Materials.

## Supplementary Materials

The supporting information can be downloaded at: https://www.mdpi.com/article/10.3390/cimb45020111/s1.
