# Supplementary material for: Quantification of the Diversity in Gene Structures Using the Principles of Polarization Mapping
Source: Curr Issues Mol Biol. 2023 Feb 18;45(2):1720–40. doi: 10.3390/cimb45020111 (PMC9955201; doi:10.3390/cimb45020111)
Supplement: Supplementary file 1 [file cimb-45-00111-s001.zip › cimb-2005913-supplementary.pdf]

current issues in molecular biology

MDPI

Supplementary Materials to “Quantification of the Diversity in Gene Structures Using the Principles of Polarization Mapping”

Dmitry Zimnyakov [1,2,3,*], Marina Alonova [1], Anatoly Skripal [3], Sergey Dobdin [3], and Valentina Feodorova[3]

[1] Yury Gagarin State Technical University of Saratov, Physics Department, 77 Polytechnicheskaya St., 410054 Saratov, Russia
[2] Precision Mechanics and Control Institute of Russian Academy of Sciences, 24 Rabochaya St., 410024, Saratov, Russia
[3] Saratov State University, Institute of Physics, 83 Astrakhanskaya St., 410012 Saratov, Russia
* Correspondence: zimnykov@mail.ru

S1. The source code for converting a fragment of the nucleotide sequence into a phase-modulating matrix (Matlab programming environment). Before converting, it is necessary to recode the original (A,C,T,G) symbol sequence into a numeric sequence in accordance with the following rule: A → 1; C → 2; T → 3; G → 4 (for example, using any text editor allowing for overall replacement of characters). The recoded file file.txt is placed in DRIVE:\DIRECTORY\, and the output file file1.txt with a phase-modulating matrix is created in 'DRIVE:\DIRECTORY1\.

```
clc; clear all;         %clearing memory

format compact
fni=(['DRIVE:\DIRECTORY\file.txt']);     %path to the processed file with a numeric sequence
A1=dlmread(fni); %reading the number sequence
A0=size(A1); %evaluation of the dataset size
for m=1:1:A0(1)   %formation of a 1D array E(S,1) of initial data
for n=1:1:A0(2)
s=(m-1)*A0(2)+n;
if A1(m,n)>0
E(s,1)=double(A1(m,n));
S=s;
end
end
end
N1=floor(sqrt(S/3)); %evaluation of the size of triplet matrix
N2=N1*2; %evaluation of the size of phase-modulating matrix
D1=zeros([N2 N2]);

%below is the fragment of the source code for generating submatrices and the final formation of the phase modulating matrix
for m=1:1:N1
for n=1:1:N1
s=3*((m-1)*N1+n)-2;
m0=2*m-1;
m1=2*m-1;
m2=2*m;
m3=2*m;
n0=2*n-1;
n1=2*n;
n2=2*n-1;
```

```
n3=2*n;


for q=0:1:2
if E(s+q,1)==1
D1(m0,n0)=D1(m0,n0)+1;
else if E(s+q,1)==2
D1(m1,n1)=D1(m1,n1)+1;
else if E(s+q,1)==3
D1(m2,n2)=D1(m2,n2)+1;
else if E(s+q,1)==4
D1(m3,n3)=D1(m3,n3)+1;
end
end
end
end


fn=(['DRIVE:\DIRECTORY1\file1.txt']); %path to the output file containing the phase-modulating matrix
dlmwrite(fn, D1, '\t'); %writing the output file
```

S2. The source code for calculating the local components of the Stokes vector in gene-based diffraction patterns (C programming language). The input file DRIVE:\\DIRECTORY1\\file1.txt is created using the above presented MatLab procedure. The output files s1.dat, s2.dat, s3.dat and s4.dat contain the local values of the Stokes vector components. The output file map.dat contains the binarized values of the local polarization states (the case of left circular polarization).

```
#include<stdio.h>
#include<stdlib.h>
#include<math.h>
#include<conio.h>
#include<dos.h>
#include<time.h>


int i,j,k,m,N;
double refer[100][100];
double scr_x[100][100];
double re_amp_x[200][200];
double im_amp_x[200][200];
double scr_y[100][100];
double re_amp_y[200][200];
double im_amp_y[200][200];
double sum1,sum2,scale;
double E_x,E_y,fi_x,fi_y,depot;
double buffer1,buffer2,buffer3;
FILE *in,*out1,*out2,*out3,*out4,*out5;
main()
{
```

```
//opening files with the input data and for the output data
in=fopen("DRIVE:\\DIRECTORY1\\file1.txt","r");
out1=fopen("DRIVE:\\DIRECTORY2\\s1.dat","w");
out2=fopen("DRIVE:\\DIRECTORY2\\s2.dat","w");
out3=fopen("DRIVE:\\DIRECTORY2\\s3.dat","w");
out4=fopen("DRIVE:\\DIRECTORY2\\s4.dat","w");
out5=fopen("DRIVE:\\DIRECTORY2\\map.dat","w");


//input of the scale factor and the size of the phase modulating matrix
printf("Input scale\n");
scanf("%lg",&scale);
printf("Input the matrix size\n");
scanf("%d",&N);


//reading the input data and forming the phase-modulating matrix
for(i=0;i<N;i++)
{
for(j=0;j<N;j++)
{
fscanf(in,"%lg\n",&depot);
refer[i][j]=depot;
}
}
for(i=0;i<N;i++)
{
for(j=0;j<N;j++)
{
scr_x[i][j]=( M_PI/2)*refer[i][j];
scr_y[i][j]=( M_PI/2)+((M_PI/2)*refer[i][j]);
}
}


//calculation of the real and imaginary parts of the x-component of diffracted field
for(i=0;i<2*N;i++)
{
for(j=0;j<2*N;j++)
{
sum1=0,sum2=0;
for(k=0;k<N;k++)
{
for(m=0;m<N;m++)
{
sum1+=cos((scale*(2*M_PI/N)*((k-((N/2)-1))*(i-N+1)+(m-((N/2)-1))*(j- N+1)))+scr_x[k][m]);
sum2+=sin(-(scale*(2*M_PI/N)*((k-((N/2)-1))*(i-N+1)+(m-((N/2)-1))*(j-N+1)))+scr_x[k][m]);
}
}
re_amp_x[i][j]=sum1/(N*N);
im_amp_x[i][j]=sum2/(N*N);
}
}
```

```
// calculation of the real and imaginary parts of the y-component of diffracted field
for(i=0;i<2*N;i++)
{
for(j=0;j<2*N;j++)
{
sum1=0,sum2=0;
for(k=0;k<N;k++)
{
for(m=0;m<N;m++)
{
sum1+=cos((scale*(2*M_PI/N)*((k-((N/2)-1))*(i-N+1)+(m-((N/2)-1))*(j-N+1)))+scr_y[k][m]);
sum2+=sin(-(scale*(2*M_PI/N)*((k-((N/2)-1))*(i-N+1)+(m-((N/2)-1))*(j-N+1)))+scr_y[k][m]);
}
}
re_amp_y[i][j]=sum1/N*N;
im_amp_y[i][j]=sum2/N*N;
}
}


//calculation and output of the local values of the Stokes vector components
for(i=0;i<140;i++)
{
for(j=0;j<140;j++)
{
E_x=sqrt(re_amp_x[i][j]*re_amp_x[i][j]+im_amp_x[i][j]*im_amp_x[i][j]);
E_y=sqrt(re_amp_y[i][j]*re_amp_y[i][j]+im_amp_y[i][j]*im_amp_y[i][j]
fi_x=atan(im_amp_x[i][j]/re_amp_x[i][j]);
fi_y=atan(im_amp_y[i][j]/re_amp_y[i][j]);

fprintf(out1,"%g\t",log10((E_x*E_x+E_y*E_y)/2));
fprintf(out2,"%g\t",(E_x*E_x-E_y*E_y)/(E_x*E_x+E_y*E_y));
buffer1=(E_x*E_x-E_y*E_y)/(E_x*E_x+E_y*E_y);
buffer2=(2*E_x*E_y*cos(fi_x-fi_y))/(E_x*E_x+E_y*E_y);
buffer3=(2*E_x*E_y*sin(fi_x-fi_y))/(E_x*E_x+E_y*E_y);
fprintf(out3,"%g\t",buffer2);
fprintf(out4,"%g\t",buffer3);


//formation of the binary map of the extreme polarization states
if(((buffer3<-0.99)&&(buffer3>-1.0)))fprintf(out5,"%g\t",1.0);
else fprintf(out5,"%g\t",0.0);
}
fprintf(out1,"\n");
fprintf(out2,"\n");
fprintf(out3,"\n");
fprintf(out4,"\n");
fprintf(out5,"\n");
}
}
```

S3. The differences in the analyzed nucleotide sequences

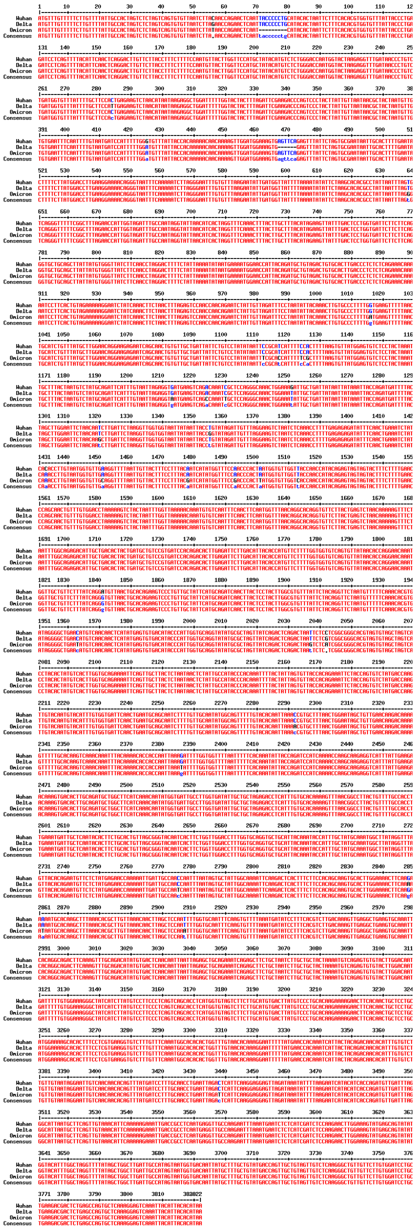

(**a**)

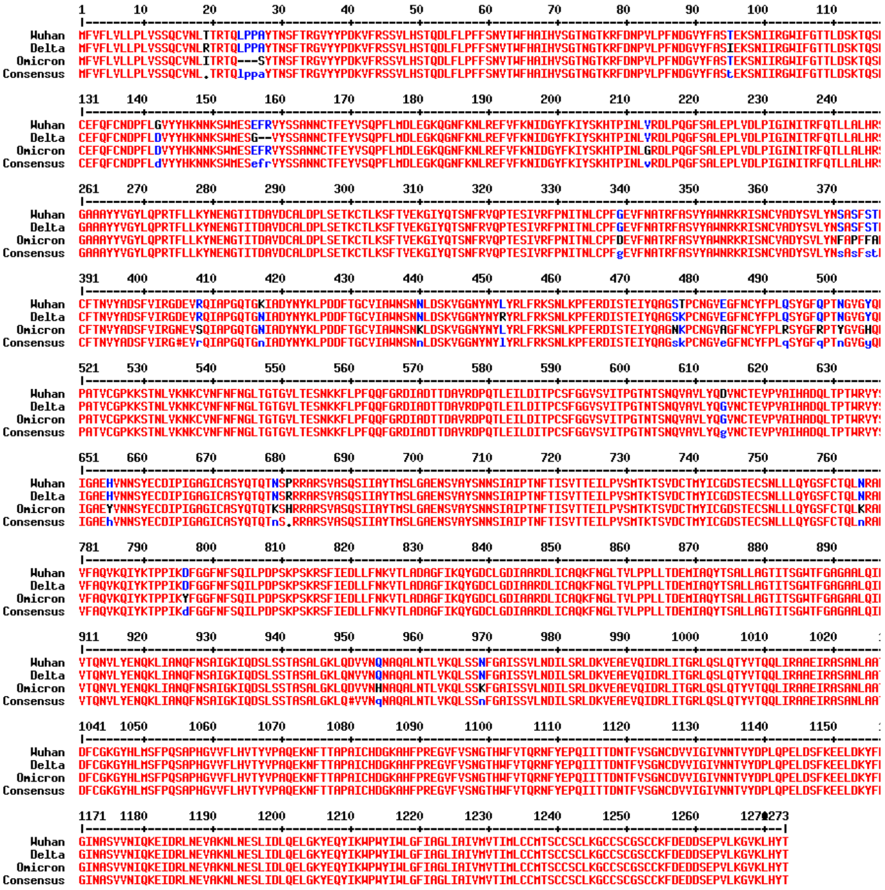

(**b**)

Figure S1. Multiple sequence alignment of the Spike gene nucleotide (a) and amino acid sequences (b) derived from the Wuhan, Delta and Omicron model strains using the Multalin online service (http://multalin.toulouse.inra.fr/multalin/). The SNPs and deletions are colored in black.

Copyright: © 2023 by the authors. Licensee MDPI, Basel, Switzerland. This article is an open access article distributed under the terms and conditions of the Creative Commons Attribution (CC BY) license (https://creativecommons.org/licenses/by/4.0/).

The differences in the nucleotide triplet sequences and corresponding amino acid substitutions in the Spike protein are presented in Table S1. The first row in the Table defines the triplet positions; triplets that differ are marked in red, while those that match

are green-highlighted. Similarly, the nonsynonymous substitutions of amino acids are red-highlighted, and the synonymous substitutions are marked in green.

Table S1. Differences (mismatches) in the nucleotide triplet sequences of the Spike gene belonging to three SARS-CoV-2 variants: Wuhan, Delta, and Omicron and corresponding amino acid substitutions

Position of the triplet	Substitutions in the either Spike gene nucleotide sequence or Spike protein of the SARS-CoV-2 variant				
	Wuhan	Delta		Omicron	
		Substitutions in the nucleotide sequence	AA Substitutions in the protein	Substitutions in the nucleotide sequence	AA Substitutions in the protein
19	ACA	AGA	T19R	ATA	T19I
95	ACT	ATT	T95I	ACT	
142	GGT	GAT	G142D	GAT	G142D
213	GTG	GTG		GGG	V213G
339	GGT	GGT		GAT	G339D
371	TCC	TCC		TTC	S371F
373	TCA	TCA		CCA	S373P
375	TCC	TCC		TTC	S375F
376	ACT	ACT		GCT	T376A
405	GAT	GAT		AAT	D405N
408	AGA	AGA		AGC	R408S
410	ATC	ATC		ATT	I410I synonymous substitution
417	AAG	AAT	K417N	AAT	K417N
440	AAT	AAT		AAG	N440K
452	CTG	CGG	L452R	CTG	
477	AGC	AGC		AAC	S477N
478	ACA	AAA	T478K	AAA	T478K
484	GAA	GAA		GCA	E484A
493	CAA	CAA		CGA	Q493R
498	CAA	CAA		CGA	Q498R
501	AAT	AAT		TAT	N501Y
505	TAC	TAC		CAC	Y505H
614	GAT	GGT	D614G	GGT	D614G
655	CAT	CAT		TAT	H655Y
679	AAT	AAT		AAG	N679K
681	CCT	CGT	P681R	CAT	P681H
764	AAC	AAC		AAA	N764K
796	GAT	GAT		TAT	D796Y
925	AAC	AAC		AAT	N925N synonymous substitution

950	GAT	**AAT**	**D950N**	**GAT**	
954	CAA	**CAA**		**CAT**	**Q954H**
969	AAT	**AAT**		**AAA**	N969K
1146	GAC	**GAC**		**GAT**	**D1146D synonymous substitution**

Disclaimer/Publisher's Note: The statements, opinions and data contained in all publications are solely those of the individual author(s) and contributor(s) and not of MDPI and/or the editor(s). MDPI and/or the editor(s) disclaim responsibility for any injury to people or property resulting from any ideas, methods, instructions or products referred to in the content.
